# Local Integral Regression Network for Cell Nuclei Detection †

**DOI:** 10.3390/e23101336

**Published:** 2021-10-14

**Authors:** Xiao Zhou, Miao Gu, Zhen Cheng

**Affiliations:** Department of Automation, Tsinghua University, Beijing 100084, China; zhouxiao17@mails.tsinghua.edu.cn (X.Z.); gum16@mails.tsinghua.edu.cn (M.G.)

**Keywords:** nuclei detection, convolutional neural networks, fully supervised learning, weakly supervised learning, local integral regression

## Abstract

Nuclei detection is a fundamental task in the field of histopathology image analysis and remains challenging due to cellular heterogeneity. Recent studies explore convolutional neural networks to either isolate them with sophisticated boundaries (segmentation-based methods) or locate the centroids of the nuclei (counting-based approaches). Although these two methods have demonstrated superior success, their fully supervised training demands considerable and laborious pixel-wise annotations manually labeled by pathology experts. To alleviate such tedious effort and reduce the annotation cost, we propose a novel local integral regression network (LIRNet) that allows both fully and weakly supervised learning (FSL/WSL) frameworks for nuclei detection. Furthermore, the LIRNet can output an exquisite density map of nuclei, in which the localization of each nucleus is barely affected by the post-processing algorithms. The quantitative experimental results demonstrate that the FSL version of the LIRNet achieves a state-of-the-art performance compared to other counterparts. In addition, the WSL version has exhibited a competitive detection performance and an effortless data annotation that requires only 17.5% of the annotation effort.

## 1. Introduction

Along with the rapid development of deep learning and computer vision, histopathology image analysis has become a centrally important research area in the field of computational pathology. The density, morphology, and distribution of cell nuclei, in the microscopic images of tissue biopsy stained with hematoxylin and eosin (H&E) or immunohistochemistry (IHC), can provide quantitative support and significant clues for assessing both the cancer grades and prognosis [1,2]. Nuclei detection is also the crucial and basic step for downstream cell classification, thus playing a critical role in computer-aided diagnosis [3]. Compared with the traditional methods that require pathologists to visually count and manually evaluate cancerous nuclei, automatic and accurate nuclei detection is highly desirable due to the increasing scale of image data [4]. The detection of cell nuclei in histopathology images is still a challenging task because the nuclei display variability of size, shape, orientation, and intensity, while the microscopic image also faces the issues of the nuclei overlapping and being out of focus due to the depth of field.

Deep learning approaches have been proven to produce encouraging results on histopathology images in various studies [5,6,7]. Recent learning-based studies on nuclei detection mainly focus on two perspectives: nuclei segmentation and nuclei counting. The segmentation-based algorithms [8,9], especially the instance segmentation, can delineate each nucleus with a sophisticated boundary under the supervision of pixel-wise annotations, which is a highly time-consuming and specialized task. The counting-based approaches [10,11], however, only require point annotation located on the center of each nucleus to generate the ground truth for network training. It outputs a density map of nuclei where the global integral and the local maxima suggest the population of nuclei and their centroids [12,13], respectively. Unfortunately, as a result of blurry maps [14] caused by the widely utilized pixel-wise Euclidean regression loss, the counting-based methods can hardly provide the precise location of each nucleus [15,16]. Moreover, even though the point annotation is a good option in terms of preserving the information and saving effort, it still requires pathologists to pinpoint the centroids of the nuclei in histopathology images [17], which is quite costly on account of the small size and the large number of cell nuclei.

To address the above-mentioned problems of counting-based approaches, a novel local integral regression network (LIRNet) for nuclei detection is proposed in this paper. Firstly, a rigorously designed loss function that restricts the local integral around each annotated point, instead of single pixel in the density map, is adopted in the proposed LIRNet. The local integral regression loss does not pre-define any hypothesis on the distribution of cell nuclei density, but still separates nuclei with clear background gaps. As a consequence, the LIRNet can both provide the evident localization of each nucleus and count the nuclei population. Secondly, since the local integral calculates the sum of the density values in a local area while ignoring the specific position of each annotated point, it is reasonable to extend the local area to artificially meshed image patches, which intuitively yields a weakly supervised learning (WSL) framework. In the WSL framework of LIRNet, the histopathology image is gridded into patches for nuclei detection. Meanwhile, pathologists only need to distinguish whether there is zero, one, or multiple nuclei in one image patch. As a result, instead of a pixel-wise centroid annotation, patch-level labels are exploited to guide the training of the WSL framework. Finally, the quantitative experimental results show that the fully supervised learning (FSL) version of the LIRNet achieves a state-of-the-art performance, and the weak annotation strategy greatly reduces the labeling cost by 82.5% on average.

In summary, the key contributions of this paper are as follows:

- We propose a novel local integral regression network that allows both fully and weakly supervised learning frameworks for estimating the conspicuous location of each nucleus in histopathology images.
- We creatively design a patch-level annotation method to reduce the annotation cost and explore a weakly supervised learning approach for nuclei detection task.
- The comparative experimental results quantitatively show that the FSL version of the LIRNet achieves a state-of-the-art performance, while the WSL version has exhibited a competitive detection ability with much less annotation cost.

The rest of this paper is organized as follows: Section 2 first introduces the current work in nuclei detection; Section 3 presents the details of the data description, annotation, and the proposed LIRNet; the experimental results and the discussion of this work are exhibited in Section 4 and Section 5; and Section 6 concludes the paper.

## 2. Related Work

Traditional nuclei detection methods rely on the extraction of hand-crafted features such as color, texture, edge, and intensity information [18,19,20]. They are designed for a certain type of histopathology images, and usually cannot work well when encountering large variations in tissue types and nuclear appearances. Distinct from these methods, learning-based methods automatically learn the high-level features of input the data. In this section, we provide an overview of the related work in two aspects: (1) learning-based nuclei segmentation and counting methods in histopathology images and (2) weakly supervised algorithms in natural and medical images.

### 2.1. Learning-Based Nuclei Segmentation and Counting

Learning-based nuclei detection can be achieved from two aspects: segmentation and counting. The segmentation-based approaches aim to predict a pixel-wise probability map of the histopathology image, in which each nucleus is supposed to be separated by clear boundaries. However, the counting-based algorithms intend to estimate a density map of cell nuclei where the local maxima indicates the predicted position of each nucleus.

Segmentation: In 2017, Kumar et al. [21] adopted a classification-based approach to distinguish the category (nuclei area or background) of each pixel in H&E stained images. Some researchers [22,23] developed watershed approaches to segment and count the cell nuclei in histopathology images. Then, Saha et al. [6] proposed a HER2 deep neural network (Her2Net) to segment and classify both the cell nuclei and membranes in breast cancer cells. Naylor et al. [8] completed the nuclei segmentation by applying a fully convolutional network to regress the distance map of the cell nuclei. Mahmood et al. [9] utilized a conditional generative adversarial network to segment multi-organ nuclei. Hou et al. [24] proposed an unsupervised learning algorithm, named a sparse convolutional autoencoder, to segment nuclei from the foreground of the cell images. Although the nuclei segmentation provides more valuable information, including the size and the morphology compared with the counting-based method, it demands more accurate pixel-wise boundary annotations.

Counting: Most of the nuclei detection studies [4,10,11,12,13,25,26,27,28] follow a regression training framework that adopts fully convolutional networks. A pseudo-density map of the nuclei is generated by infusing Gaussian-like distribution masks around an individual annotated point as the ground truth. Some early studies [15,16,29] adopted unsupervised learning or the nuclei population in image patches to predict a counting value as well as the density map. Khan et al. [5] proposed a classification-based method to count human embryonic cells. However, those approaches can hardly provide the precise location of an individual nucleus without the supervision of point annotations [15,16]. Afterwards, Xie et al. [7] trained a multi-task framework to provide both a location vector and a confidence score for each pixel. Rad et al. [30,31] developed a residual dilated U-net to count and locate each human embryo. Generally, the traditional counting-based approaches demand a pseudo-density map as the ground truth, which unfortunately inclines to trigger a blurred effect on sharp edges.

### 2.2. Weakly Supervised Learning

Fully supervised object localization requires bounding box labels or point annotations, which suffers from costly manual annotation. Accordingly, many studies [32,33,34,35,36,37] exploit weakly supervised object localization that only requires image-level labels for the network training. Class activation mapping (CAM) [32] adopts a full convolutional network to yield a score map predictor before the global average pooling layer. Afterwards, diverse network architectures [33,36] and data-augmentation strategies [34,35] are proposed to cover the whole object region. However, few studies use weakly supervised learning to localize a small instance, especially in histopathology images. Recently, Qu et al. [17] proposed a weakly supervised nuclei segmentation algorithm which utilized partial point annotations in each training image as the ground truth, while simpler patch-level labels are required in our WSL framework.

## 3. Materials and Methods

### 3.1. Dataset Description

**MBM cells:** To assess the contribution of each part of our approach, we adopt the Modified Bone Marrow (MBM) dataset [16,38] that consists of 44 H&E stained images with 126±33 cells per image from healthy individuals. After the standard staining procedure, the purple blue depicts the nuclei of the various cell types, whereas the other cell constituents appear in various shades of pink and red. In our experiments, 16 and 6 images are randomly selected as the training and validation dataset. The rest of the 22 images act as the test samples.

**CA cells:** To evaluate the performance of our WSL algorithm and compare the proposed FSL framework with state-of-the-art counterparts, we also employ the colorectal adenocarcinoma (CA) dataset [4] containing 100 H&E stained histopathology images with 29,756 nuclei annotated in total. It represents real-world challenges, for instance, overlapping nuclei and background interference, and is widely used in recent deep learning-based nuclei detection algorithms. Instead of the conventional point annotations, the ground truth for the WSL framework training is yielded by gridding the histopathology images into patches and then labeling them with counting indicators, which is described in the data annotation. The dataset is randomly divided into training, validation, and test sets with a ratio of 7:1:2.

**PSU cells:** Additionally, a dataset [39] from Penn State University (PSU) with fluorescently labeled cell nuclei is applied to evaluate the nuclei detection performance. The PSU dataset includes 120 images of colon tissues from 12 pigs with a total number of 25,462 nuclei, in which an 80:20:20 split is employed for the training, validation, and test sets. It only visualizes cell nuclei with 4′,6-diamidino-2-phenylindole (DAPI) and, meanwhile, comprises areas with over-staining and failed auto focusing to represent outliers normally found in real scenarios.

### 3.2. Dataset Annotation

**Figure 1:**
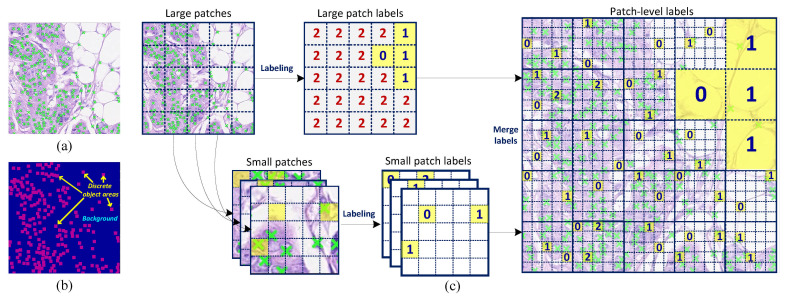
Data annotation for different learning strategies. (**a**) The point annotations of nuclei in a histopathology image, in which the green crosses mark the center of each nucleus. (**b**) The ground truth of a fully supervised learning (FSL) framework can be divided into two parts: discrete object areas (red pixels) and a merged background region (blue pixels). (**c**) The patch-level annotation procedure for the weakly supervised learning (WSL) framework. A histopathology image is firstly divided into large patches and labeled with the truncated counting indicators. Subsequently, the large patches with the indicator I=2 are further gridded into small patches. To reduce the annotation cost, only part of the small patches are randomly chosen and labeled with the truncated counting indicators.

Two different data annotation strategies are applied to the proposed LIRNet for nuclei detection. On one hand, the fully supervised learning framework requires all of the point annotation centered at each nucleus (Figure 1a) to yield object areas (red pixels) and a merged background area (pixels in blue), as shown in Figure 1b. On the other hand, the histopathology images are divided into patches in the weakly supervised learning framework, and a truncated counting indicator I={0,1,2} is designed to label each image patch which separately represents zero, one, and at least two nuclei. As a result, pathologists are not demanded to either pinpoint their centroids or count a precise population of nuclei in the data labeling procedure. Instead, they only need to make a simple ternary judgment on whether there is a nucleus and, if yes, whether there is one nucleus or more than one nucleus in each divided patch.

Considering that the nuclei are not uniformly distributed in most histopathology images (with high-density nuclei) which causes a label imbalance between I={0,1} and I=2, a further gridding on the dense-nucleus (I=2) patches is executed to obtain more detailed labels. In this work, a histopathology image is firstly divided into large patches with the resolution of 100×100 and labeled with the truncated counting indicators I. Subsequently, the large patches with the indicator I=2 are further gridded into small patches with the resolution of 20×20. To reduce the annotation cost, only part of the small patches, rather than all of them, are randomly chosen and further labeled with the truncated counting indicators. Figure 1c shows the schematic diagram of data annotation for the WSL framework.

### 3.3. Network Architecture of LIRNet

**Figure 2:**
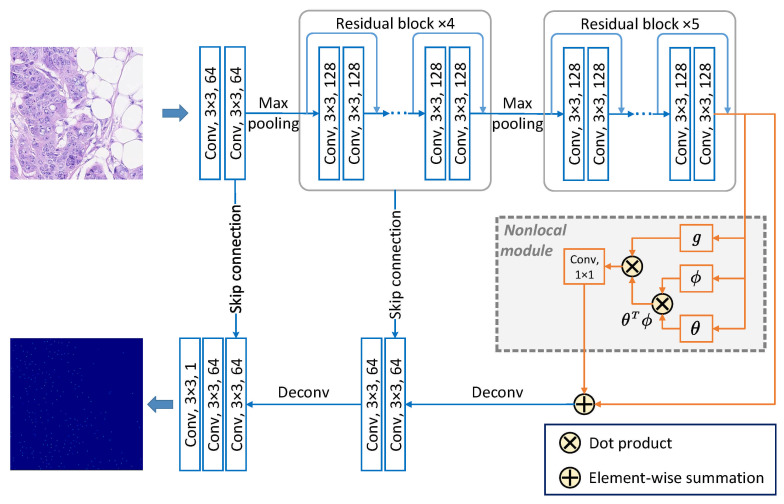
The architecture of the local integral regression network (LIRNet), in which nine residual blocks and a nonlocal module are separately inserted into the feature extraction layers and the bottom layer of a lightweight U-net.

The schematic diagram of the LIRNet architecture is shown in Figure 2. As a typical example of fully convolutional networks, U-net [40] is widely applied due to its extraordinary performance on the segmentation of medical images. In this paper, we develop a lightweight U-net, with nine residual blocks from residual networks (ResNets) [41] inserted into the feature extraction layers, to predict a density map of cell nuclei which shares the same image resolution with the input image data.

Besides, a nonlocal module [42] is introduced into the bottom layer of the lightweight U-net to facilitate the localization of each nucleus. Acting as an extension of self-attention [43], the nonlocal module is usually used to extract global features and explore nonlocal relationships among nonadjacent positions. Generally, each type of nucleus in microscopic histopathology images shares limited variants of cell morphology, which can be explored and highlighted by inserting a nonlocal module into the whole network. The dot product is adopted as the pairwise function of the nonlocal module to learn the appearance similarity of the nuclei. The rectified linear unit (ReLU) [44] is utilized as the activation function in the whole LIRNet network.

### 3.4. Loss Function Design

#### 3.4.1. Full Supervision

Unlike the continuously distributed density map employed in traditional counting-based approaches [12,13,27], the density map in this work is divided into two areas: discrete object areas, remarked by {$O_{k}|k=1,2,\ldots ,N$}, and a merged background area B, as shown in Figure 1b. Consequently, we propose a novel local integral regression loss function that consists of two parts: local integral object loss and local integral background loss. Taking into consideration that the integral of the whole density map indicates the nuclei population, it is reasonable to assume that the local integral of the nucleus density around an annotated point equals to 1 even though there is no hypothesis about its density distribution. With respect to the background loss, the integral of the density value in any local background area is supposed to be 0, which can not only isolate an individual nucleus by a clear boundary but also guarantee the whole nuclei population. Nevertheless, the background area is a continuous region that can hardly be divided into separate islets. Therefore, we utilize the mean integral of the background pixels to approximate the local integral. Ultimately, an old version of the overall loss function under the full supervision in our previous work [45] can be written as:

(1) $$L_{LIR}^{f}\;=\;\lambda \;\frac{1}{N_{O}}\sum_{k\;=\;1}^{N_{O}}{\left(\sum_{\left(i,j\right)\in O_{k}}D_{ij}-1\right)}^{2}\;+\;\left(1-\lambda \right)\;\;\tanh\left(\frac{S_{O}}{S_{B}}\sum_{\left(m,n\right)\in B\;\;}D_{mn}\right)$$

where the first and the second items separately represent the object loss and the background loss. D denotes the output density map, $O_{k}$ and $N_{O}$ suggest the *k*th object area and the number of discrete object areas, respectively. $S_{O}$ and $S_{B}$ individually describe the number of pixels in an object area and the whole background. λ serves as a weight coefficient to balance the object loss with the background loss, which is a hyperparameter to be manually adjusted.

While in this work, we abandon the hyperparameter λ in the interest of conciseness and further replace each loss item with $\ell_{1}$ form. Finally, the overall loss function of the FSL algorithm is formulated by:

(2) $$L_{\ell_{1}LIR}^{f}\;=\;\sum_{k\;=\;1}^{N_{O}}\left|\sum_{\left(i,j\right)\in O_{k}}D_{ij}-1\right|+\left|\sum_{\left(m,n\right)\in B\;\;}D_{mn}\right|$$

We find that the $\ell_{1}$ loss without any hyperparameters outperforms the previous loss in Equation (1), which is demonstrated in the Results section. Meanwhile, to test the superiority of the proposed local integral regression loss, a widely used control experiment that adopts a pixel-wise Euclidean regression error [13] is also introduced as a baseline, which is written as:

(3) $$L_{PER}^{f}\;=\;{∥D\;\;-\;\;D_{gt}∥}_{F}^{2}$$

where D and $D_{gt}$ represent the output density map and the true density map, respectively. The true density map is generated by embedding a Gaussian distribution at each annotated point.

#### 3.4.2. Weak Supervision

As described in the data annotation and Figure 1c, the ground truth for the WSL framework is made up of patch-level counting indicators I. We now define $I_{k}$ and $C_{k}^{P}\;=\;\sum_{\left(i,j\right)\in P_{k}}D_{ij}$ that denote the annotated counting indicator and the counting prediction of the *k*th image patch $P_{k}$, respectively. Then, taking advantage of the mean square error (MSE) to construct the loss function between $I_{k}$ and $C_{k}^{P}$ is intuitively practical. However, because the counting indicator $I_{k}\;=\;2$ represents a dense-nuclei patch with at least 2 nuclei instead of a precise population, the traditional MSE loss will indiscriminately compel all of the corresponding counting predictions to approach 2. Accordingly, even if the true number of nuclei in an image patch might be much greater than 2, especially for the large patches, at most, 2 nuclei can be precisely detected. In order to address this underestimate problem, a truncation function is designed to truncate the counting prediction of multi-nucleus patches, which can be formulated by:

(4) $$T\left(C_{k}^{P}\right)\;=\;\left\{\begin{array}{cc} C_{k}^{P} & C_{k}^{P}<2\\ 2+\gamma C_{k}^{P} & C_{k}^{P}⩾2 \end{array}\right.$$

where γ represents a positive constant that provides a small gradient designed to avoid the vanishing gradient issue. As a result, the loss function for each type of indicator $I_{k}$ can be constructed by the $\ell_{2}$ form and an optimized hyperbolic tangent function [45]:

(5) $$L^{k}\;=\;\left\{\begin{array}{c} \begin{array}{cc} \tanh\left(T\left(C_{k}^{P}\right)\right) & I_{k}\;=\;0\\ {\left(T\left(C_{k}^{P}\right)-1\right)}^{2} & I_{k}\;=\;1 \end{array}\\ \begin{array}{cc} \tanh\left(2-T\left(C_{k}^{P}\right)\right) & I_{k}\;=\;2 \end{array} \end{array}\right.$$

In this work, we also replace each loss item mentioned above with the $\ell_{1}$ form for the same reason, hence the former Equation (5) is rewritten as:

(6) $$L_{\ell_{1}}^{k}\;=\;\left\{\begin{array}{cc} \left|I_{k}-T\left(C_{k}^{P}\right)\right| & I_{k}\;=\;0,1\\ I_{k}-T\left(C_{k}^{P}\right) & I_{k}\;=\;2 \end{array}\right.$$

It should be noted that there is no |·| operating on the item for the indicator $I_{k}=2$. It is mainly because this patch $P_{k}$ has more than 2 nuclei, and the truncated counting prediction is expected to surpass its indicator value 2. Even so, it can not guarantee a reasonable end because the above loss for $I_{k}=2$ in Equations (5) and (6) is a monotonic decreasing function. Consequently, a regularization term on the counting prediction is necessary to avoid the obviously counterintuitive results. Since the LIRNet outputs a density map of the nuclei with the same resolution as the input image, it is reasonable to assume that the density value of each pixel in the density map is less than 1. For the reason that the limitation on the density value of each pixel is equivalent to the restriction on the max density value in the density map, the regularization term can thus be formulated by:

(7) $$\psi \left(D\right)\;=\;max\left(D_{max}-1,0\right)$$

where $D_{max}$ represents the max value in the density map. Consequently, the whole loss function of the WSL algorithm, including the previous truncated combine loss and the $\ell_{1}$ form in this work, can be individually formulated by:

(8) $$L_{TC}^{w}\;=\;\frac{1}{N_{P}}\sum_{k=1}^{N_{P}}L^{k}+\;\eta \psi \left(D\right)$$

(9) $$L_{\ell_{1}TC}^{w}\;=\;\frac{1}{N_{P}}\sum_{k=1}^{N_{P}}L_{\ell_{1}}^{k}+\;\eta \psi \left(D\right)$$

where η is the weight of the regularization term. $N_{P}$ denotes the number of training patches.

### 3.5. Nuclei Localization

Post-processing algorithms on the density map, which could be predicted by the the LIRNet, are desired to obtain the accurate location of an individual nucleus. According to the traditional experience of counting-based approaches [12,13], the integral of the density map and the local maxima of the resulting image are identified as the counting prediction and the central positions of nuclei, respectively. Therefore, by heuristically searching the top *N* local maxima on the density map, where *N* denotes the integral of the predicted density map, the nuclei locations can be obtained. A simplified version of non-maximum suppression (NMS) [46] is adopted to reduce false positives. It directly erases all of the density values in a circular neighborhood with a radius *r* centered at each local maximum.

### 3.6. Evaluation Metrics

We adopt the common metrics, including precision (*P*), recall (*R*), and F1 score (F1), to quantify the nuclei detection performance of our algorithm and other competitive supervised approaches.

(10) $$P=\frac{TP}{TP+FP},\;\;R=\frac{TP}{TP+FN},\;\;F1=\frac{2PR}{P+R}$$

Same as Sirinukunwattana et al. [4], Zhou et al. [10], and Tofighi et al. [39], a circular area centered at each annotated nucleus centroid is regarded as the golden standard region. The radius of the circular neighborhood is set to 6 pixels for the CA/MBM cell dataset and 10 pixels for the PSU cell dataset. Accordingly, a detected nucleus is judged to be a true positive (TP) if its predicted location is nearest to an annotated nucleus centroid and falls into its golden standard region. In case of multiple detected nuclei around the same ground truth point, only the closest one is considered as a true positive. The ground truth points which have no corresponding detection are false negatives (FN). The same “Golden Region” is used across all of the methods that are in comparison.

To measure the localization accuracy of each method, we also adopt the median, 1st quartile (Q1), and 3rd quartile (Q3) of the distribution of the Euclidean distance between each detected nucleus and its nearest annotated center of nucleus.

### 3.7. Training Details and Implementation

The object area for local integral is generated by a square kernel centered at an individual annotated point. The size of the square kernel is set according to the average distance between each nucleus and its 2 nearest neighbors (to reduce the influence of repeat labeling on the same nucleus) in the training dataset. As a result, the kernel size is set to 15, 11, and 13 pixels for the MBM, CA, and PSU datasets, respectively, which is exhibited in Table 1. The small constant γ in the truncation function Equation (4) and the coefficient of the regularization η in Equation (9) are set to 0.0001 and 0.001, respectively. The configuration of the weight λ in Equation (1) is set uniformly to 0.1 in all of the experiments.

Data augmentations, including rotation and flipping, are conducted on the images to promote the accuracy of location. Besides, both the original image and its augmented form are simultaneously fed into the network at each training iteration, which is conceived to promote the detection performance. The training is optimized by the Adam optimizer [47] with a batch size of 12 (6 original + 6 augmented) for 200 epochs. The learning rate is initialized with 0.0001 and adjusted by the F1 score of the validation set. The model that achieves the best performance in the validation dataset is recorded as the final model.

## 4. Results

We first evaluate the performance of the FSL version of the proposed approach and compare it with state-of-the-art counterparts. Afterwards, the performance and the ability to reduce the annotation cost of the WSL framework are extensively evaluated on the CA and MBM datasets. Finally, we quantitatively assess the contribution of each part of our algorithm.

### 4.1. Comparison with Counterparts

We compare our LIRNet with eight competitive approaches on the CA cells dataset, including stacked sparse autoencoder (SSAE) [29], local isotropic phase symmetry measure (LIPSyM) [48], symmetric residual convolutional neural network (SR-CNN) [25], spatially constrained convolutional neural network (SC-CNN) [4], shape prior convolutional neural network (SP-CNN) [49], sibling fully convolutional network with prior objectness interaction (SFCN-OPI) [10], vector oriented confidence accumulation (VOCA) [7], and shape prior convolutional neural network (TSP-CNN) [39]. Specifically, LIPSyM exploits the morphological features of nuclei to detect their centroids in H&E stained images. SSAE adopts an unsupervised learning framework to identify the high-level features of nuclei, while both SR-CNN and SC-CNN apply a ConvNet to estimate the density map of nuclei under the full supervision of point annotations. SP-CNN and its updated version, tunable SP-CNN (TSP-CNN), take advantage of additional prior-guided shape information to enhance nuclei detection. SFCN-OPI completes the nuclei detection and classification by applying a unified framework and achieves the best recall. VOCA develops a multi-task learning framework to provide both a location vector and a confidence score for each pixel. These methods are chosen because they are widely compared.

The quantitative results of the comparative performance are shown in Table 2, in which LIR and LIR-$\ell_{1}$ separately represent the proposed LIRNet with the $\ell_{2}$-tanh [45] and $\ell_{1}$ loss function. The experimental results of all of the counterparts are derived from their papers [4,7,10,39]. The NA means the results were not reported and not available by that method. Although SFCN-OPI detects 87.4% nuclei, our LIRNet with $\ell_{1}$ loss achieves both the best precision at 0.864 and the highest F1 score of 0.858. The quantitative gains in precision, the F1 score, and the Q3 metric are quite explicit in Table 2. The comparison of the Q1/Q3 metrics illustrates that the distances between 25%/75% of detected nuclei and their nearest annotated points are within 1.414/3.606 pixels, which suggests that the LIRNet can largely promote the location accuracy of nuclei detection.

**Figure 3:**
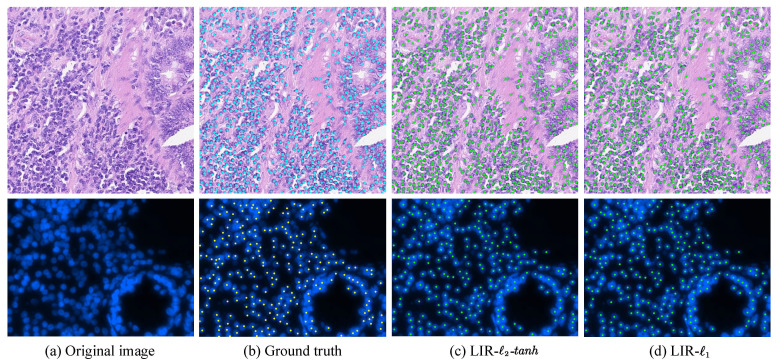
Typical results of the FSL framework for nuclei detection on the CA (up) and PSU (down) cell datasets. The F1 scores of our method LIR-$\ell_{2}$-tanh on the two images are 0.899 (up) and 0.905 (down), respectively. The F1 scores of LIR-$\ell_{1}$ on the two images are 0.919 (up) and 0.912 (down), respectively.

We also evaluate the proposed FSL framework on the PSU dataset [39], and the performance comparison is reported in Table 3. It can be seen that the LIR-$\ell_{2}$-tanh achieves the best precision, and its $\ell_{1}$ form achieves the second-best F1 score, which is 0.011 lower than that of the TSP-CNN. This is largely because the additional information, including the cell nuclei shapes, is utilized as trainable prior knowledge during the training procedure in the TSP-CNN [39]. Nevertheless, both LIR-$\ell_{2}$-tanh and LIR-$\ell_{1}$ of the LIRNet achieve a better performance than the SP-CNN, which is the previous version of the TSP-CNN with fixed shape priors. Figure 3 visually exhibits typical results of nuclei detection on both the CA and PSU datasets. It could be observed that nearly all of the nuclei are captured by the LIR-$\ell_{2}$-tanh method, and the LIR-$\ell_{1}$ approach further improves the detection performance.

### 4.2. Weakly Supervised Learning Results

We count and compare the cost of both the proposed patch-level labeling and the conventional point annotations, so as to measure the capability of our WSL framework on reducing the data annotation cost. It could be quantified by the number of mouse clicks. The experimental results on the CA dataset are illustrated in Table 4. According to the statistical analysis, the average number of mouse clicks for the conventional point annotations is about 280 per image in the training dataset. In contrast, a different number of patch-level labels are set to quantify the performance of our WSL method. In our previous study [45], it is achieved by randomly labeling five small patches in a different number of large patches with the counting indicator I=2. While in this work, we randomly annotate a different number of small patches from all of the large patches with the counting indicator I=2, as shown in Figure 1c.

Applying the former annotation strategy, at most, 130 patch-level labels per image are provided on average, which reduces the annotation cost by at least 53.5%. As a result of the trade-off, the F1 sore of the WSL with $L_{TC}^{w}$ loss is decreased by 0.06 compared with the FSL version. While for the latter annotation strategy with new $L_{\ell_{1}TC}^{w}$ loss, the F1 sore of the WSL is still above 0.8 when 122 patch-level labels are provided on average, which outperforms other fully supervised approaches, such as SR-CNN and SC-CNN (Table 2), and simultaneously reduces the annotation cost by 56.4%. As the number of annotated labels continues to decrease, the mean annotation costs of both 98 and 74 patch labels are reduced to 35% and 26.4% that of fully supervised training, respectively. Meanwhile, the F1 scores of the WSL with $L_{\ell_{1}TC}^{w}$ loss are only reduced by 0.007 and 0.03 compared with the situation that 122 patch-level labels are provided. It clearly reveals that our WSL LIRNet algorithm possesses both an effortless data annotation and a competitive performance of nuclei detection. Similar trends can also be seen in $L_{TC}^{w}$ loss in terms of the benefits of patch-level labeling. Even though 49 patch-level labels (17.5% of total annotation cost) are provided in each image, the F1 performance of the WSL with $L_{\ell_{1}TC}^{w}$ loss can still remain above 0.75, which is close to 72 labels of the WSL with $L_{TC}^{w}$ loss.

The WSL results on the MBM dataset are exhibited in Table 5. It is also obvious that the achieved F1 score is above 0.8 even though less than half of the annotated labels are provided. The above comparison results demonstrate that our WSL algorithm can achieve a competitive performance of nuclei detection while largely reducing the annotation cost. More importantly, in the aspect of the labeling complexity, the point annotation requires precise clicks in the centroid pixel of each nucleus one by one. Nevertheless, the patch-level labeling only demands one judgmental click on each image patch, which is obviously more flexible and convenient when compared with traditional point annotation.

### 4.3. Ablation Study

#### 4.3.1. Contribution of Nonlocal Module

To verify the ability of a nonlocal module on extracting global features of cell morphology and exploring the relationships among different positions, we first evaluate and compare the detection performance of different network variants. The experimental results on both the MBM and CA cells dataset are exhibited in Table 6, in which $N^{+}$ and $N^{-}$ individually suggest the whole network with and without a nonlocal module. It could be concluded that the network variants ($N^{+}$, $L_{LIR}^{f}$) achieve a higher F1 score than the network variants ($N^{-}$, $L_{LIR}^{f}$) on both datasets, clearly confirming the effectiveness of the nonlocal module.

**Figure 4:**
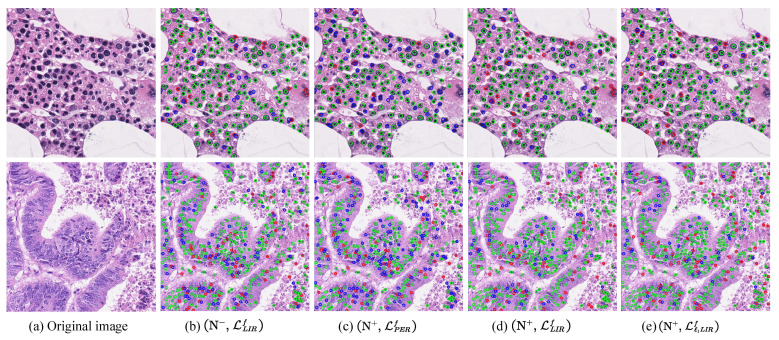
Typical detection results of ablation study from the MBM cell (first row) and CA cell (second row) datasets. Green, blue, and red circles represent ground truth with correct detection (TP), ground truth without correct detection (FN), and false positive detection (FP), respectively. The F1 score for ($N^{-}$, $L_{LIR}^{f}$), ($N^{+}$, $L_{PER}^{f}$), ($N^{+}$, $L_{LIR}^{f}$), and ($N^{+}$, $L_{\ell_{1}LIR}^{f}$) in the first row are 0.874, 0.806, 0.875, and 0.884, respectively. The F1 score in the second row are 0.798, 0.720, 0.855, and 0.856, respectively. More clearly displayed in color and enlargement.

Figure 4 provides further insight into the merits of different network variants on two typical images from the MBM and CA datasets. Due to the evidently distinguishable nuclei morphology on the MBM dataset (first row), the network variant ($N^{-}$, $L_{LIR}^{f}$) and the ($N^{+}$, $L_{LIR}^{f}$) achieves nearly the same detection performance. However, on the CA dataset (second row), the morphological features of the cells are substantially diverse even though they are located adjacently. As a result, the network variant ($N^{-}$, $L_{LIR}^{f}$) fails to localize a large number of nuclei that distributed at the middle, left, and bottom of the image, while the network with a nonlocal module ($N^{+}$, $L_{LIR}^{f}$) significantly reduces false negatives (FN, blue circles) and thus increases the F1 score by 0.057. This improved performance demonstrates that the nonlocal module can enhance the network to explore the morphological similarity and further apply it to forward reasoning.

#### 4.3.2. Contribution of Local Integral Regression Loss

In order to explore the effectiveness of the loss function design, we perform an ablation study between the local integral regression (LIR) loss (Equations (1) and (2)) and the pixel-wise Euclidean regression loss (Equation (3)). Table 6 shows the performance comparison among the $L_{LIR}^{f}$, $L_{PER}^{f}$, and $L_{\ell_{1}LIR}^{f}$ loss. It is obvious that the F1 score of the variants ($N^{+}$, $L_{LIR}^{f}$) separately achieve 7.8% and 5.1% higher than that of the variants ($N^{+}$, $L_{PER}^{f}$) on the MBM and the CA datasets. Besides, the $\ell_{1}$ form of LIR loss ($L_{\ell_{1}LIR}^{f}$) achieves the best performance over all of the network variants, suggesting that $L_{\ell_{1}LIR}^{f}$ is the most appropriate loss function for the LIRNet training procedure.

Figure 4c–e exhibits the comparison of nuclei detection among different network variants, including the network with pixel-wise Euclidean regression loss ($N^{+}$, $L_{PER}^{f}$) and the networks with our LIR loss ($N^{+}$, $L_{LIR}^{f}$), ($N^{+}$, $L_{\ell_{1}LIR}^{f}$). It can be observed that ($N^{+}$, $L_{PER}^{f}$) indeed omits a large amount of cell nuclei (blue circles) on both of the datasets, while the variant ($N^{+}$, $L_{LIR}^{f}$) with LIR loss significantly reduces false negatives and increases the F1 score by 6.9% and 13.5% on the MBM and CA images, respectively. Besides, the detection performance is further improved by the network with the updating LIR loss in the $\ell_{1}$ form, as shown in Figure 4 and Table 6.

**Figure 5:**
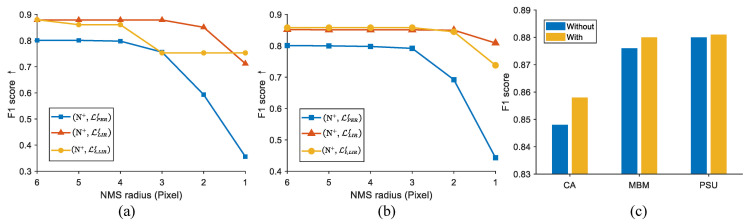
(**a**,**b**) The comparison of detection performance under different non-maximum suppression (NMS) radii in the MBM cell and the CA cell datasets, respectively. (**c**) Performance comparison with and without data augmentation strategy.

Since our approach adopts the tanh and $\ell_{1}$ functions to generate the background loss and provide constraints, the LIRNet can output a more elaborate density map of the nuclei with much less background noise. As a consequence, the network with LIR loss is less sensitive towards the radius of the circular neighborhood in NMS than the $L_{PER}^{f}$ network variant. Figure 5a,b displays the variation of detection performance with the change of the NMS radius. It can be seen that the F1 score of the $L_{PER}^{f}$ network variant deteriorates sharply as the NMS radius decreases. In contrast, the F1 score of the variants with LIR loss still remain above 0.7 even though the true positive is restricted within one pixel from the true annotated point, which reaches a fairly strict standard. In a brief, the LIRNet predicts a refined density map of the nuclei in which nuclei localization is barely affected by the post-processing algorithms.

#### 4.3.3. Contribution of Data Augmentation Strategy

In the training stage, both the original image and its randomly augmented form (rotation/flipping) are simultaneously fed into the network at each iteration. To exhibit its contribution, we also compare the performance with and without employing this training strategy on the ($N^{+}$, $L_{\ell_{1}LIR}^{f}$) network. Figure 5c shows the experimental results on three datasets. It can be observed that all of the achieved F1 scores with the data augmentation strategy, which improves the robustness of the localization, are higher than those without the strategy. In spite of the limited benefit on the PSU dataset, the data augmentation strategy increases the performance by nearly 1% on the CA dataset. The insignificant improvement on the PSU dataset is mainly because the background and the morphology in the DAPI-labeled cells are clearly distinguishable, which weakens the effect of the data augmentation strategy.

## 5. Discussion

We manually set each object area to the uniform square kernel according to the average distance between two nuclei in the histopathology images, as shown in Table 1, to reduce the difficulty of computing the local integral. A significant direction for future work is to provide an adaptive size for each detected object, especially for the cells with different morphology.

In addition, we attempt to explain how the weakly supervised nuclei detection works in three aspects. First of all, the positions of the nuclei and the specific numbers remain unknown for the WSL training procedure. By comparing empty patches with single-nucleus ones, the LIRNet manages to distinguish and localize the nucleus from the background. Secondly, both single-nucleus and multi-nucleus patches provide weak counting supervision for the proposed network. Therefore, the LIRNet is capable of learning to count the number of nuclei, particularly for the regions with high-density nuclei. Thirdly, the nonlocal module in the LIRNet can effectively spread the learned capability of nuclei localization to the multi-nucleus patches on account of the similarity in appearance of nuclei with identical morphology.

## 6. Conclusions

In this paper, we propose a novel local integral regression network that allows both fully and weakly supervised learning frameworks on nuclei detection. Compared with other fully supervised learning approaches, the FSL version of LIRNet achieves state-of-the-art detection performance, while the WSL version of LIRNet possesses a competitive detection performance and an effortless data annotation that requires much less annotation effort. Prospectively, the proposed approaches could offer a benefit to pathology practice in terms of a quantitative analysis of tissue images, and potentially lead to a better understanding of disease. Besides, considering the fact that LIRNet promotes the precision of location, it can conceivably be extended to other computer vision applications, for instance, the crowd detection issues.

## Figures and Tables

**Table 1:** The configuration of square kernel size. The distance between each nucleus and its 2 nearest neighbors in the training dataset, including Modified Bone Marrow (MBM), colorectal adenocarcinoma (CA) and labeled nuclei from Penn State University (PSU), is calculated.

	MBM Cells	CA Cells	PSU Cells
Distance	15.22 ± 3.51	10.34 ± 6.03	12.56 ± 1.48
Configuration	15	11	13

**Table 2:** The comparison of nuclei detection performance among different approaches on the CA dataset. Bold and blue font suggest the best and the second-best performance, respectively. NA indicates the result were not available. LIR-$\ell_{2}$-tanh and LIR-$\ell_{1}$ represent our approach with $\ell_{2}$-tanh loss (Equation (1)) and $\ell_{1}$ loss (Equation (2)). ↑/↓ means higher/lower is better.

Method	Precision ↑	Recall ↑	F1 ↑	Median Distance ↓ (Pixels)	(Q1, Q3) ↓ (Pixels)
SSAE	0.617	0.644	0.630	4.123	(2.236, 10)
LIPSyM	0.725	0.517	0.604	**2.236**	(**1.414**, 7.211)
SR-CNN	0.783	0.804	0.793	**2.236**	(**1.414**, 5)
SC-CNN	0.781	0.823	0.802	**2.236**	(**1.414**, 5)
SP-CNN	0.803	0.843	0.823	NA	NA
SFCN-OPI	0.819	**0.874**	0.834	NA	NA
VOCA	0.831	0.863	0.847	2.0 *	(1.414, 2.236) *
TSP-CNN	0.848	0.857	0.852	NA	NA
LIR-$\ell_{2}$-tanh	0.854	0.850	0.852	**2.236**	(**1.414**, **3.162**)
LIR-$\ell_{1}$	**0.864**	0.852	**0.858**	**2.236**	(**1.414**, 3.606)

* This method evaluates the Euclidean distance between each pair of ground truth and its assigned detection.

**Table 3:** The comparison of nuclei detection performance on the PSU dataset. Bold and blue font suggest the best and the second-best performance, respectively. LIR-$\ell_{2}$-tanh and LIR-$\ell_{1}$ represent our approach with $\ell_{2}$-tanh loss (Equation (1)) and $\ell_{1}$ loss (Equation (2)). ↑/↓ means higher/lower is better.

Method	Precision ↑	Recall ↑	F1 ↑
SSAE	0.665	0.634	0.649
SR-CNN	0.797	0.805	0.801
SC-CNN	0.821	0.830	0.825
SP-CNN	0.854	0.871	0.863
TSP-CNN	0.874	**0.911**	**0.892**
LIR-$\ell_{2}$-tanh	**0.875**	0.871	0.873
LIR-$\ell_{1}$	0.869	0.893	0.881

**Table 4:** Experimental results of the WSL framework on the CA cell dataset. Underline and bold font represent the best performance of the FSL and WSL framework, respectively. Blue font suggests the minimal labels provided in the WSL framework.

	Method	Loss	Number of Labels (Ratio%)	Precision ↑	Recall ↑	F1 ↑	Median Distance (Q1,Q3) ↓ (Pixels)
Previous work	FSL	$L_{LIR}^{f}$	280 (100%)	0.854	0.850	0.852	2.236 (1.414, 3.162)
	WSL	$L_{TC}^{w}$	130 (46.4%)	**0.810**	0.777	**0.793**	3.162 (2.236, **5.0**)
	WSL	$L_{TC}^{w}$	92 (32.9%)	0.773	**0.792**	0.783	**3.0** (2.236, 5.099)
	WSL	$L_{TC}^{w}$	72 (25.7%)	0.772	0.739	0.755	3.162 (**2.0**, 5.099)
This paper	FSL	$L_{\ell_{1}LIR}^{f}$	280 (100%)	0.864	0.852	0.858	2.236 (1.414, 3.606)
	WSL	$L_{\ell_{1}TC}^{w}$	122 (43.6%)	0.790	0.823	**0.807**	2.828 (**1.414**, 4.472)
	WSL	$L_{\ell_{1}TC}^{w}$	98 (35.0%)	**0.809**	0.791	0.800	**2.236** (**1.414**, **4.123**)
	WSL	$L_{\ell_{1}TC}^{w}$	74 (26.4%)	0.730	**0.830**	0.777	2.828 (**1.414**, 6.0)
	WSL	$L_{\ell_{1}TC}^{w}$	49 (17.5%)	0.747	0.758	0.753	3.0 (2.0, 5.385)

**Table 5:** Experimental results of the WSL framework on the MBM cell dataset. Underline and bold font represent the best performance of the FSL and WSL framework, respectively. Blue font suggests the minimal labels provided in the WSL framework.

Method	Loss	Number of Labels (Ratio%)	Precision ↑	Recall ↑	F1 ↑	Median Distance (Q1,Q3) ↓ (Pixels)
FSL	$L_{\ell_{1}LIR}^{f}$	129 (100%)	0.867	0.893	0.880	2.828 (2.000, 4.123)
WSL	$L_{\ell_{1}TC}^{w}$	86 (66.7%)	**0.893**	**0.812**	**0.825**	3.162 (**2.0, 5.0**)
WSL	$L_{\ell_{1}TC}^{w}$	61 (47.3%)	0.808	0.798	0.803	**3.0 (2.0, 5.0)**
WSL	$L_{\ell_{1}TC}^{w}$	51 (39.5%)	0.707	0.738	0.722	3.606 (2.236, 6.083)

**Table 6:** Experimental results of ablation study on the MBM cell and CA cell datasets. $N^{+}$ and $N^{-}$ suggest the whole network with and without a nonlocal module, respectively. Bold font suggests the best performance.

Dataset	Network Variants	Precision ↑	Recall ↑	F1 ↑
MBM cells	($N^{-}$, $L_{LIR}^{f}$)	0.877	0.874	0.875
	($N^{+}$, $L_{PER}^{f}$)	**0.905**	0.718	0.801
	($N^{+}$, $L_{LIR}^{f}$)	0.885	0.873	0.879
	($N^{+}$, $L_{\ell_{1}LIR}^{f}$)	0.867	**0.893**	**0.880**
CA cells	($N^{-}$, $L_{LIR}^{f}$)	0.862	0.811	0.836
	($N^{+}$, $L_{PER}^{f}$)	0.848	0.759	0.801
	($N^{+}$, $L_{LIR}^{f}$)	0.854	0.850	0.852
	($N^{+}$, $L_{\ell_{1}LIR}^{f}$)	**0.864**	**0.852**	**0.858**

## Data Availability

In this work, we exploited the publicly available MBM dataset [16,38], CA dataset [4], and PSU dataset [39]. The code is available on GitHub at the following link https://github.com/Astaxanthin/LIRNet (accessed on 1 October 2021).
